# Functional Role of Class III Myosins in Hair Cells

**DOI:** 10.3389/fcell.2021.643856

**Published:** 2021-02-25

**Authors:** Joseph A. Cirilo, Laura K. Gunther, Christopher M. Yengo

**Affiliations:** Department of Cellular and Molecular Physiology, College of Medicine, Pennsylvania State University, Hershey, PA, United States

**Keywords:** actin, myosin, ATPase, stereocilia, MYO3A, MYO3B, force generation

## Abstract

Cytoskeletal motors produce force and motion using the energy from ATP hydrolysis and function in a variety of mechanical roles in cells including muscle contraction, cargo transport, and cell division. Actin-based myosin motors have been shown to play crucial roles in the development and function of the stereocilia of auditory and vestibular inner ear hair cells. Hair cells can contain hundreds of stereocilia, which rely on myosin motors to elongate, organize, and stabilize their structure. Mutations in many stereocilia-associated myosins have been shown to cause hearing loss in both humans and animal models suggesting that each myosin isoform has a specific function in these unique parallel actin bundle-based protrusions. Here we review what is known about the classes of myosins that function in the stereocilia, with a special focus on class III myosins that harbor point mutations associated with delayed onset hearing loss. Much has been learned about the role of the two class III myosin isoforms, MYO3A and MYO3B, in maintaining the precise stereocilia lengths required for normal hearing. We propose a model for how class III myosins play a key role in regulating stereocilia lengths and demonstrate how their motor and regulatory properties are particularly well suited for this function. We conclude that ongoing studies on class III myosins and other stereocilia-associated myosins are extremely important and may lead to novel therapeutic strategies for the treatment of hearing loss due to stereocilia degeneration.

## Introduction

**FIGURE 1:**
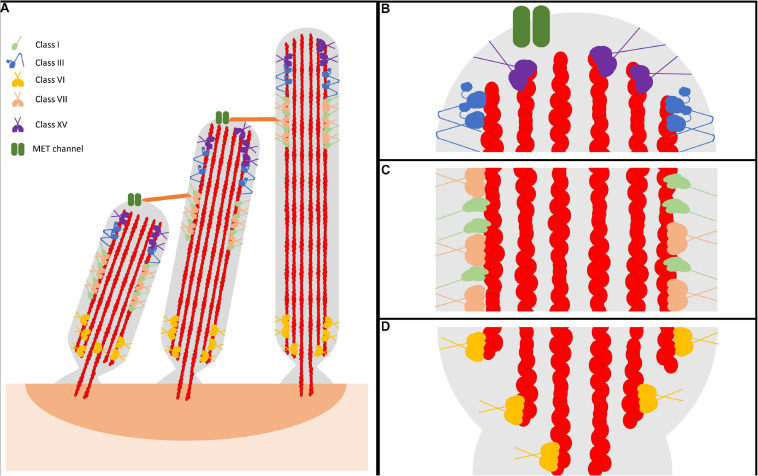
Representative model of myosin motor localization in inner ear hair cell stereocilia. **(A)** Three rows of stereocilia form a staircase-like pattern, tethered together by various tip links, that is maintained through an organism’s lifetime. In an individual stereocilium, there are three main regions where myosins localize: **(B)** the stereocilia tips, also known as the lower tip link density, containing class III myosins and class XV myosins, as well as MET channels, **(C)** the lower tip link density, containing class I and class VII myosins, and **(D)** the anklet, containing class VI myosins. MYO7A has been shown to localize throughout the entire length of the stereocilia, however since its proposed function as a tensioning myosin occurs at the UTLD it is shown only here for simplicity. Apart from class III myosins, all stereocilia myosins have direct evidence of membrane binding. However, class III myosins are hypothesized to interact with the membrane, potentially modulated via its binding partner MORN4. Lastly, both MYO7A and MYO15A have not been directly shown to form dimers on their own, however MYO7A can form dimers via a cargo-mediated mechanism and MYO15A was proposed to oligmerize in a complex with cargo. For simplicity, both are represented as dimers.

Hearing is a complex physiological processes that ultimately requires the conversion of a mechanical signal, in the form of sound waves, into an electrical signal that can be recognized by the auditory cortex in the brain. Unfortunately, when this tightly regulated process is disrupted, various forms of hearing loss can occur. In general, hearing loss affects 2 out of every 1,000 infants, with half of those cases having a genetic origin (DiStefano et al., 2019). In total, mutations in over 100 genes have been proposed to be associated with non-syndromic hearing loss and another 400 associated with syndromic hearing loss. The high prevalence of genetic forms of hearing loss highlights the importance of understanding the molecular causes and mechanisms of disease pathogenesis. Auditory and vestibular inner ear hair cells are the sensory cells of the inner ear that contain mechanosensitive cellular protrusions called stereocilia (White et al., 2020). In the organ of Corti, which houses the auditory inner ear hair cells, the stereocilia form a defined staircase-like pattern comprised of three rows of ascending lengths (Figure 1A). Each stereocilia is connected to its smaller/larger neighbor via a tip link, consisting of cadherin-23 and protcadherin-15 (Siemens et al., 2002; Kazmierczak et al., 2007; Grati et al., 2016). Also found at the tip are mechanoelectrical transduction, or MET, channels that respond to mechanical changes in the stereocilia, and ultimately trigger the electrophysiological response produced by the inner ear hair cell (Corey et al., 2019). Recent work has identified two subunits of the MET complex found at stereocilia tips, TMC1 and TMC2, which likely function as the mechanosenstive channels in stereocilia (Kurima et al., 2015; Yue et al., 2019; Jia et al., 2020). Specifically, as stereocilia are displaced by sound waves, the increased tension on MET channels increases their open probability and allows for the influx of cations into the hair cell, which causes a graded electrophysiological response relative to the degree of stereocilia displacement (Fettiplace, 2017). The stereocilia then can undergo a process known as adaptation, whereby they rapidly reset, leading to channel closing in preparation for the next stimulus. Depolarization of hair cells leads to an increase in synaptic release onto the afferent auditory neurons. Stereocilia length and morphology are critical for normal mechanotransduction, and therefore disruption of either of these properties results in hearing loss (Tilney and Saunders, 1983; Vélez-Ortega and Frolenkov, 2019).

Stereocilia are composed of a cytoskeleton actin core that is tightly bundled and crosslinked, providing a strong scaffold for the cellular protrusion (Tilney et al., 1980; Rzadzinska et al., 2005; Figure 1). The crosslinked actin filaments in the stereocilia are aligned parallel to each other with their plus (barbed) ends orientated at the tips. There are three main regions within the stereocilia: the tip, the tip-link, and the anklet. At the stereocilia tips, the actin cytoskeleton is thought to be more dynamic, undergoing regular turnover, while the remaining actin at the shaft and base is extremely stable (Zhang et al., 2012; McGrath et al., 2017; Vélez-Ortega and Frolenkov, 2019). The upper tip link density, located along the stereocilia shaft, contains complexes of proteins responsible for maintaining the link between adjacent stereocilia (Grati and Kachar, 2011; He et al., 2019). Lastly, the anklet contains strong links between the actin cytoskeleton, which is tapered at the base, and the membrane and helps maintain stereocilia morphology at the base (Grati et al., 2012). Though the tips are dynamic, stereocilia length must be precisely maintained throughout an organism’s lifetime. A key question in the field is how stereocilia length is maintained both throughout development, as well as in a mature organism. In addressing this question, recent studies have determined that the turnover of actin at the tips in adult hair cells is restricted only to the tip region and requires actin severing proteins, suggesting a unique mechanism of actin turnover separate from what is observed in more dynamic actin protrusions (Zhang et al., 2012; Drummond et al., 2015; Narayanan et al., 2015). For example, filopodia and microvilli are known to renew their actin bundles by an actin treadmilling mechanism, a process by which new actin monomers are added to the protrusion tips and travel to the base as they cycle through the filament (Tyska and Mooseker, 2002; Loomis et al., 2003; Craig et al., 2012). In addition, researchers have identified many different stereocilia-associated proteins found to play essential roles in formation, morphology, and maintenance. Amongst these proteins are various myosin motors, including the tip localizing MYO3A/B and MYO15A (Figure 1B), the tip-link associated MYO7A and MYO1C (Figure 1C), and the ankle tethering MYO6 (Figure 1D; Peng et al., 2011). Each motor is proposed to be important for different aspects of stereocilia morphology/function and thus loss of any individual myosin motor leads to stereocilia degeneration and hearing loss (Lin et al., 2011; Rehman et al., 2016; Dantas et al., 2018; Calabro et al., 2019; Wang et al., 2019). Therefore, numerous studies have examined stereocilia-associated myosins providing crucial insight into their role in the hearing process.

Photoreceptors contain actin-based protrusions called the calyceal processes, though their function is currently unknown (Nagle et al., 1986; Dosé et al., 2003). Both MYO7A and MYO3A/B, as well as the stereocilia-associated proteins protocadherin-15, cadherin-23, whirlin, sans, and harmonin were all found to be localized to the calyceal processes (Hasson et al., 1995; Dosé et al., 2003; Kremer et al., 2006; Lelli et al., 2016). Mutations in any of these proteins, with the exception of MYO3A/B, cause a form of deafness and blindness called Usher syndrome (Hasson et al., 1995; Kremer et al., 2006). Overall, the structure of stereocilia and calyceal processes is similar, however the stereocilia are maintained throughout a lifetime while the calyceal processes are renewed regularly (1–6 weeks) (Young, 1967; Schietroma et al., 2017). Thus, myosins may have a parallel function in these similar actin-based structures that are found in two critically important sensory cells.

## The Myosin Superfamily

**FIGURE 2:**
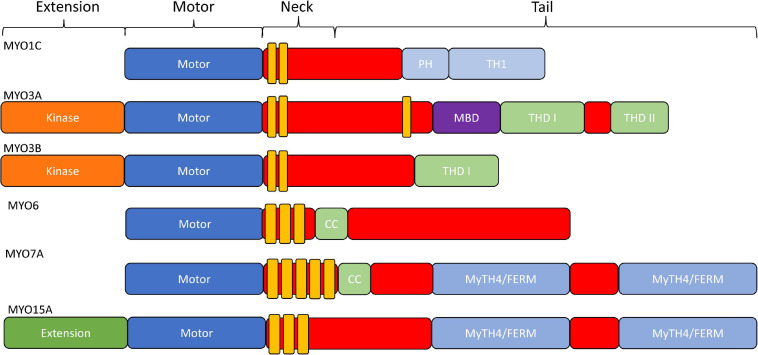
Domain structure of stereocilia-associated myosins. In general, all myosins contain a conserved N-terminal motor domain (head), light chain binding region (neck), and a variable C-terminal domain (tail) which allows specialized functions (e.g., dimerization, filament formation, membrane binding, and cargo/adapter protein binding). Additionally, MYO1C has a membrane binding Pleckstrin-Homology domain, as well as a membrane binding tail homology domain. MYO3A/MYO3B, MYO6, and MYO15A all contain an N-terminal extension domain, with the MYO3A/MYO3B extension being a protein kinase. MYO6 and MYO7A have dimerizing coiled-coil domains, and MYO7A and MYO15A each contain two membrane binding MyTH4/FERM domains.

The typical domain structure of myosins is composed of an N-terminal conserved motor domain (head), a light chain binding region (neck), and a class specific C-terminal region domain (tail) (Figure 2; Heissler and Sellers, 2016). The motor domain contains the ATP and actin binding regions, which is allosterically connected to the light chain binding neck referred to as the lever arm. When myosin is strongly bound to actin the lever arm tilts to allow force generation or translocation along actin (Houdusse and Sweeney, 2016). The light chain binding neck is comprised of a variable number of IQ (consensus sequence: [IVL]QXXXRXXXX[RK]XX[FILVWY]) motifs that bind to calmodulin or calmodulin like proteins (Rhoads and Friedberg, 1997). The tail domain is the most variable region of myosins. For example, the tail domain can contain a coiled-coil for dimerization, a filament forming region, or cargo/membrane binding motifs (Heissler and Sellers, 2016). Myosins such as MYO3A/B and MYO15A contain unique N-terminal extensions that have been proposed to alter motor activity (Dosé et al., 2008). Thus, it is postulated that all myosins utilize their conserved motor domain to produce mechanical work, while the variable tail domain allows myosins to localize to specific regions of the cell, carry specific cargo, or assemble into contractile filaments.

**FIGURE 3:**
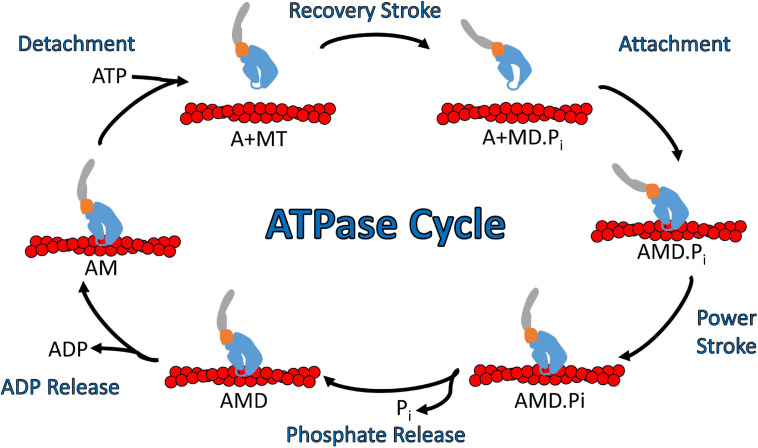
Diagram of the actomyosin ATPase cycle. Myosin cycles between “weak” actin binding and “strong” actin binding states and while strongly bound generates a power stroke that drives filament sliding or walking along actin. Specifically, ATP binding to myosin dissociates it from actin which is followed by ATP hydrolysis and a transition into the pre-power stroke state (recovery stroke). Myosin binding to actin with the hydrolyzed products in its active site leads to strong actin binding, the myosin power stroke, and the release of phosphate. After the power stroke, ADP is released from the nucleotide pocket in a strong actin binding state, which allows for another ATP molecule to bind and start a new ATPase cycle. Abbreviations for actin, myosin, ATP, ADP, ADP.Pi are A, M, T, D, D.Pi, respectively.

In general, myosins are P-loop associated ATPases with structural similarities to G-proteins and kinesins (Kull et al., 1998). The acto-myosin ATPase cycle is well conserved within the myosin superfamily, while the rate and equilibrium constants that control the cycle are unique to each myosin which allows them to perform specific cellular functions (Figure 3; De La Cruz and Ostap, 2004; Geeves, 2016; Heissler and Sellers, 2016). Myosin binds tightly to actin in the absence of nucleotide. ATP binds to the nucleotide binding region via several conserved nucleotide-binding elements: P-loop, switch I, and switch II (Kull et al., 1998). Once ATP binds, there is a dramatic weakening of the affinity of myosin for actin. The hydrolysis of ATP occurs while myosin is detached from actin and allows the lever arm to be positioned in a pre-power stroke conformation (recovery stroke). When myosin with the hydrolyzed products in the active site binds to actin there is a transition into the strongly bound actomyosin state and a shift from a pre-power stroke to a post-power stroke state that is key to force generation (power stroke) as well as the acceleration of phosphate release (Trivedi et al., 2015; Gunther et al., 2020). The release of ADP from actomyosin is required before another ATP cycle can occur. Individual steps of the ATPase cycle (e.g., ATP binding, ATP hydrolysis, ADP release etc.) can be analyzed via transient kinetic stopped-flow analysis (De La Cruz and Ostap, 2009). An important parameter that can be determined from kinetic analysis is the myosin duty ratio, or the fraction of the ATPase cycle that myosin spends tightly bound to actin (De La Cruz and Ostap, 2004; O’Connell et al., 2007). Myosins that demonstrate a high duty ratio (≥0.5) can function as a processive dimeric motor, capable of taking multiple steps along actin without diffusing away from the track, and thus are well suited to function as cargo transporters (Moore et al., 2001). On the other hand, low duty ratio motors typically function in teams to generate ensemble force and motion by interacting with actin filaments.

Myosins have been the subject of biomedical research for quite some time, with various members of the superfamily being associated with a wide array of disease conditions (Trivedi et al., 2020). For example, mutations in *MYH7*, the gene that encodes beta-cardiac myosin, have been shown to lead to various types of cardiomyopathies, including hypertrophic and dilated cardiomyopathy (Yotti et al., 2019). Similarly, mutations in the *MYO5A* gene, that encodes the cargo-transporting MYO5A, have been associated with Griscelli’s Syndrome, a debilitating neurological disorder (Emanuel et al., 2007). It is important to understand how these mutations alter the conserved mechanism of force generation in myosins. Recently, small molecule allosteric regulators of myosin have been successfully developed to treat heart disease, suggesting this strategy may be successful for treating other myosin-associated disease conditions (Malik et al., 2011; Green et al., 2016; Alsulami and Marston, 2020).

### Myosins and Deafness

Various classes of myosin have been found to localize to different parts of the stereocilia and be involved in the hearing process. Class I myosins are small monomeric myosin motors that share a typical function of linking the membrane and actin cytoskeleton (McIntosh and Ostap, 2016). Two class I myosins have been described to play potential roles in the hearing process, MYO1A and MYO1C. Although originally found expressed in the inner ear, MYO1A was later ruled out as a candidate hearing loss gene (Patton et al., 2016). Therefore, much research has focused on MYO1C as the predominant hearing-associated class I myosin (Adamek et al., 2011; Lin et al., 2011; Pyrpassopoulos et al., 2012). MYO1C, first described as the adaptation motor, localizes near the MET channels in the upper tip link density as well as at stereocilia tips (Dumont et al., 2002; Phillips et al., 2006). Within its tail domain, it contains two membrane binding domains; a Pleckstrin homology (PH) domain, and a tail homology (TH) domain (Figure 2; Hokanson et al., 2006; Chung et al., 2019). Although its role has yet to be fully elucidated, researchers have demonstrated that class I myosins have a unique strain-sensitive ADP-release mechanism (Laakso et al., 2008), which could make MYO1C ideally suited to sense the membrane tension associated with stereocilia movement (Batters et al., 2004; Laakso et al., 2008). Indeed, MYO1C was found to be crucial in MET channel adaptation, a process by which the MET channel gating is reset between stimuli (Holt et al., 2002). The adaptation process was originally thought to be regulated via MYO1C alone, though further research has suggested other myosin motors may play a role (Caprara et al., 2020).

The *MYO6* gene, found on chromosome 6, encodes for a unique unconventional myosin that walks toward the minus end of actin filaments (Avraham et al., 1997). All myosins characterized thus far, with the exception of MYO6, move toward the plus end of actin filaments (Wells et al., 1999). MYO6 contains an N-terminal canonical myosin motor followed by a single calmodulin-binding IQ motif, resulting in a short lever arm (Figure 2). However, one study suggests that there may be another calmodulin binding site found within the motor domain, affecting motor activity (Chevreux et al., 2005). Following the IQ motif, there is a three-helix bundle and a single alpha-helical (SAH) domain, both of which are predicted to contribute to cargo binding abilities (Figure 2). There is a unique 53aa insertion in the motor that is not seen in other members of the myosin superfamily. This insertion is proposed to function as the “reverse gear” and responsible for facilitating movement of the lever arm during the power stroke in the opposite direction as canonical myosins (Ménétrey et al., 2005). Also, MYO6 has been shown to bind PIP_2_ lipids in the cell membrane through an R/K rich domain in its cargo binding tail domain (Buss and Kendrick-Jones, 2008).

Within the stereocilia, MYO6 localizes at the base of the actin protrusion near the rootlet region (Seki et al., 2017), where the minus (pointed) ends of actin filaments are located (Tilney et al., 1980). Mutations in *MYO6* have been shown to be associated with both an autosomal dominant and an autosomal recessive form of hearing loss, DFNA22 and DFNB37, respectively (Melchionda et al., 2001; Ahmed et al., 2003). A mouse model that displays loss of MYO6, known as Snell’s waltzer, demonstrates a phenotype associated with balance and hearing defects including circling, head tossing, and deafness (Avraham et al., 1995; Self et al., 1999). Stereocilia lacking MYO6 were found to develop abnormally, specifically by fusing at their base and forming large stereocilia, suggesting a role for MYO6 in anchoring the stereocilia membrane to the actin core. Another study suggested that the loss of MYO6 was associated with increased levels of hair cell death (Avraham et al., 1995). Mutations in *MYO6* associated with autosomal dominant non-syndromic hearing loss (ADNSHL) have also been identified (Melchionda et al., 2001). One common mutation, R205Q, occurs in the motor domain and slows both actin-activated ATPase activity and *in vitro* motility (Kwon et al., 2014). The R205Q mutation results in a progressive hearing loss phenotype that starts within the first decade of life (Kwon et al., 2014). Other mutations that affect cargo binding, such as the truncation mutant R1166X, affect MYO6 cellular localization (Arden et al., 2016).

*MYO7A* is one of two class VII myosins within the human genome. Found on chromosome 11, the *MYO7A* gene encodes for a 250 kDa unconventional myosin that contains an N-terminal canonical myosin motor domain with a high duty ratio, five light-chain binding IQ motifs, and two MyTH4/FERM domains in its tail region, separated by an SH3 domain (Figure 2; El-Amraoui et al., 2008; Heissler and Manstein, 2012). Due to its expression in both the calyceal processes and stereocilia, MYO7A mutations have been associated with both vision and hearing loss (Calabro et al., 2019). Two distinct modes of hearing loss are commonly associated with *MYO7A* mutations, known as DFNA11 and DFNB2. DFNA11 is characterized by a progressive form of hearing loss and DFNB2 is an autosomal recessive form of hearing loss (Bakhchane et al., 2017; Yamamoto et al., 2020) that develops as early as 7 months (Riazuddin et al., 2008; Hildebrand et al., 2010). Usher syndrome type B, or USH1B, is the most common disease resulting from *MYO7A* mutations, which is characterized by both loss of vision and profound deafness. A mouse model of USH1B, known as shaker-1, contains a mutation in the *MYO7A* gene and displays both vision and hearing loss phenotypes (Eudy and Sumegi, 1999). Interestingly, *MYO7A*^(–/–)^ mice with no MYO7A expression have profound hearing and vision loss, displaying a loss of both inner and outer hair cells, though no loss in calyceal process structure or photoreceptor cells (Calabro et al., 2019).

Within the stereocilia, MYO7A can be found localized to the plasma membrane along the shaft of the protrusion, with high localization near the UTLD (Sakai et al., 2011). It is here that MYO7A associates with the tip link complex, composed of cadherin-23, protocadherin-15, sans, and harmonin. MYO7A cargo binding is proposed to trigger MYO7A dimerization and translocation to the tips of stereocilia. The tip localized MYO7A may also function by pulling on the tip-link complex, thus creating tension that has been proposed to both help regulate MET channel gating, as well as provide a strong parallel link between adjacent stereocilia (Siemens et al., 2002; Bahloul et al., 2010; Grati and Kachar, 2011; Yu et al., 2017). Interestingly, this function correlates with the role that MYO7B plays within microvilli of the intestine, where MYO7B both links the actin cytoskeleton to the membrane via its MyTH4/FERM domains as well as links adjacent villi together via its interaction with USH1C and ANKS4B (He et al., 2019). MYO7B was found to have a high duty ratio and kinetic properties that make it well suited to function as a transporter and/or mechanosensitive motor important for sensing tension on tip-link complexes that connect adjacent actin protrusions (Henn and De La Cruz, 2005; He et al., 2019). However, its slow ensemble and single molecule motility (16 and 11 nm/s, respectively) suggest it may not be well suiting for long range transport (Inoue and Ikebe, 2003; Sato et al., 2017). Two recent studies which studied the role of myosin motors in the MET channel adaptation reported that MYO7A is a good candidate to maintain resting tension on the MET channel, but its slow motility speed likely precludes it from functioning in adaptation (Caprara et al., 2020; Li et al., 2020). Thus, future studies are crucial for specifically examining the role of MYO7A in maintaining tip-link tension and MET channel gating.

MYO15A and MYO15B are two class XV myosins found on chromosome 17 (Friedman et al., 1995; Boger et al., 2001). MYO15B, was long believed to be a pseudo gene due to the absence of conserved residues known to be crucial for myosin motor domain function (Boger et al., 2001). Interestingly, proteomic analysis from this same study revealed expression of MYO15B in the stomach, kidney, and colon, though its function is yet to be elucidated. MYO15A, is unique in that it contains an N-terminal extension that is followed by a functional canonical myosin motor domain and 3 IQ motifs in the neck region (Figure 2). Similar to other unconventional myosins, MYO15A also contains two MyTH4/FERMs, allowing it to interact with the cell membrane. Mutations in both the motor domain, as well as the MyTH4/FERM domain, have been shown to cause non-syndromic hearing loss (DFNB3) (Zhang et al., 2019). Mouse models that lack MYO15A expression, including the shaker-2 mouse, show defects in both stereocilia elongation, as well as morphology (e.g., thickness), demonstrating MYO15A is required for stereocilia formation (Probst et al., 1998).

Early studies of MYO15A found that it localizes to the extreme tip of stereocilia, leading to the initial hypothesis that it was involved in the stereocilia elongation process (Belyantseva et al., 2003). Further data has shown that MYO15A also acts as a transporter, bringing the protein whirlin to the tips of stereocilia (Belyantseva et al., 2005; Delprat et al., 2005). Interestingly, whirler mice that lack a functional whirlin protein demonstrate short stereocilia and a deafness phenotype, mirroring that of shaker-2 mice. In addition, a study of transfected MYO15A in shaker-2 mouse hair cells showed both restoration of normal hair bundle morphology, as well as proper whirlin localization, further suggesting that both MYO15A and whirlin localization are critical for actin protrusion elongation (Belyantseva et al., 2005). Furthermore, studies that utilized a MYO15A isoform specific KO mouse showed that stereocilia bundles that expressed only the short isoform of MYO15A, which lacks the N-terminal extension, would initially develop normally, but they would retract as early as P11, with a near full retraction occurring by P50 (Fang et al., 2015). However, the long MYO15A isoform is able to traffic to these bundles and prevent disassembly, suggesting a role in stereocilia architecture and maintenance. Recent kinetic analysis of the MYO15A motor demonstrated a moderate duty ratio (∼0.5) and relatively high affinity for actin (Jiang et al., 2021). *In vitro* motility experiments demonstrated that MYO15A can move actin filaments at rate of ∼300–400 nm/s and confocal microscopy showed localization of MYO15A at protrusion tips of live cells (Belyantseva et al., 2005; Bird et al., 2014). There are two possible models for MYO15A that can explain its ability to act as a transporter within stereocilia. One such model suggests that MYO15A functions as a dimer or oligomer, perhaps as a complex with cargo, to move processively toward the actin protrusion tips (Jiang et al., 2021). The second model suggests a membrane binding mediated movement, whereby monomeric MYO15A associates with both the actin cytoskeleton and the plasma membrane, moving via a diffusive sliding mechanism (Bird et al., 2014). Overall, the current results suggest MYO15A functions as a tip-directed cargo transporter and/or elongation regulator.

### Class III Myosins

Class III myosins exist in two different isoforms, MYO3A and MYO3B, found on chromosomes 10 and 2, respectively (Dosé et al., 2003). Both MYO3A and MYO3B have been shown to be localized to the calyceal processes of photoreceptors as well as in the stereocilia of vestibular and auditory inner ear hair cells (Dosé et al., 2003; Schneider et al., 2006). Similar to MYO15A, both MYO3A and MYO3B localize to the tips of stereocilia. However, MYO3A and MYO3B show a “thimble-like” localization at the tips (Schneider et al., 2006), whereas MYO15A localizes at the extreme tips (Belyantseva et al., 2003). MYO3A has been shown to be associated with a form of non-syndromic hearing loss known as DFNB30 (Walsh et al., 2002, 2011). DFNB30 is a late onset form of hearing loss, with the average onset in the third or fourth decade. The localization of class III myosins at the tips of filopodia in heterologous cell culture systems correlates with the *in vivo* localization within stereocilia, which has allowed researchers to investigate binding partners and requirements for tip localization (Les Erickson, 2003; Schneider et al., 2006; Salles et al., 2009; Quintero et al., 2010; Mecklenburg et al., 2015). The overall structure of MYO3A and MYO3B are similar. Both MYO3A and MYO3B have an N-terminal kinase domain located before their canonical myosin motor and a 2-IQ neck region (Figure 2; Dosé et al., 2003). Also, both share a domain in their tail region, known as tail homology domain I (THDI) that binds the actin crosslinking protein espin (ESPN-1 and ESPN-L) via the ankyrin repeat domains (ARD) (Salles et al., 2009; Merritt et al., 2012; Ebrahim et al., 2016; Liu et al., 2016). MYO3A contains a second tail homology domain, THDII, which contains an actin binding motif known to be required for MYO3A associated tip localization (Dosé et al., 2003; Les Erickson, 2003). MYO3B lacks THDII and is thus unable to localize to protrusion tips, while fusing THDII to the MYO3B tail rescues tip localization (Merritt et al., 2012; Raval et al., 2016). Interestingly, MYO3B is thought to utilize the actin binding motif of ESPN (associated with the tail of MYO3B) to provide a second actin binding site and localization to the protrusion tips. ESPN is important for the maintenance of structures that contain tightly packed, parallel actin bundles such as microvilli and stereocilia (Loomis et al., 2003). In a cell culture model, it was shown that both MYO3A and MYO3B have the capability to transport ESPN to the tips of filopodia, though ESPN itself is necessary for MYO3B tip localization (Salles et al., 2009; Merritt et al., 2012).

MYO3A also contains a region in its tail capable of binding the membrane occupation and recognition nexus repeat containing protein, MORN4 (Mecklenburg et al., 2015; Li et al., 2019), a protein with no known function in inner ear hair cells. Similarly, cell biological studies have shown that MYO3A can co-localize with MORN4 at filopodia tips (Mecklenburg et al., 2015) while MYO3A is not necessary for MORN4 localization at stereocilia tips (Lelli et al., 2016). MORN4 is the human homolog of the *Drosophila* protein retinophilin (RTP), which can co-localize to actin-based structures in photoreceptors in *Drosophila* and directly bind to NINAC, the Drosophila homologue of class III myosins (Hicks and Williams, 1992; Porter et al., 1992; Venkatachalam et al., 2010). MYO3A contains a region in its tail between its third IQ domain and THDI where MORN4 binds (Mecklenburg et al., 2015; Li et al., 2019). The function of this interaction is still unknown, however RTP helps stabilize NINAC in *Drosophila* rhabdomeres and MORN repeat containing proteins are known to both stabilize proteins, as well as contribute to protein-membrane interactions (Mecklenburg et al., 2015). Thus, it is interesting to speculate that MORN4 serves to tether MYO3A to the plasma membrane at the tips of stereocilia and calyceal processes, while future studies in inner ear hair cells and photoreceptors are required to investigate this interesting possibility.

Studies have examined class III myosin function using a variety of approaches, including performing biochemical analysis with purified proteins and examining cellular function in heterologous cell culture systems (Dosé et al., 2007; Merritt et al., 2012; Raval et al., 2016). The motor properties of MYO3A and MYO3B are relatively well defined, with MYO3B demonstrating a twofold slower maximum ATPase rate and much weaker actin binding affinity compared to MYO3A (Merritt et al., 2012; Raval et al., 2016). Early studies of MYO3A analyzed the motor properties in a construct that contained the MYO3A kinase, motor, and neck region (including 2IQ domains), but lacking the entire tail (Dosé et al., 2007). These studies found MYO3A to have a relatively slow maximum ATPase rate (∼1 s^–1^). In addition, the same study found MYO3A to have a relatively high affinity for actin in co-sedimentation assays, as well as fast phosphate release and slow ADP release, suggesting a moderately high duty ratio for MYO3A (Dosé et al., 2007). Many of the studies that have investigated the biochemical and cell biological properties of class III myosins were performed with the kinase domain deleted (ΔK) to prevent autophosphorylation of the motor (see kinase regulation below). However, removal of the kinase domain in MYO3A enhances the maximum ATPase activity twofold (∼2 s^–1^) and increases actin affinity, which suggests the kinase domain does alter MYO3A motor properties (Dosé et al., 2008). In addition, the rate-limiting step in the MYO3A ΔK 2IQ construct was proposed to be ATP hydrolysis, but determining the actual ATP hydrolysis rate constant was challenging since ATP-binding to the MYO3A motor is extremely slow. Thus, our current kinetic simulations that assume rate limiting ATP hydrolysis suggest that the duty ratio of MYO3A ΔK 2IQ is ∼0.25, while further measurements are necessary in the kinase-containing construct. Interestingly, MYO3A ΔK 2IQ can slide filaments in the *in vitro* motility assay twofold faster than MYO3B ΔK 2IQ (71 and 31 nm/s, respectively), demonstrating it not only has faster ATPase kinetics but faster motile activity (Raval et al., 2016).

A unique property of class III myosins is their N-terminal kinase domain. Both the MYO3A and MYO3B kinase domain belong to the STE20 class of protein kinases (Quintero et al., 2013). Studies of MYO3A found two threonines in the motor domain and two in the kinase domain that are targets for intermolecular kinase domain phosphorylation (Quintero et al., 2013). Phosphorylation of the kinase domain increases kinase activity and phosphorylation of the motor domain decreases motor activity (Quintero et al., 2010, 2013). This contributes to the concentration-dependent regulation by autophosphorylation mechanism of MYO3A. In short, MYO3A is able to walk toward the tips of actin protrusions. However, once MYO3A begins to accumulate at the tip, there is an increased probability that the kinase domain of these tip localized MYO3A molecules will phosphorylate both the motor and kinase domain of other MYO3A molecules. Phosphorylation of the kinase domain increases kinase activity and phosphorylation of the motor domain decreases MYO3A motor ATPase activity and actin affinity, resulting in a higher probability of being recycled back to the cell body (Quintero et al., 2010, 2013). Therefore, the proposed auto-regulatory mechanism precisely controls MYO3A concentration at the protrusions tips which may be crucial for regulating actin protrusion lengths.

*In vivo* models of class III myosins have demonstrated their essential function within inner ear hair cells, especially during development. Specifically, previous work has shown that co-localization of MYO3A/B with ESPN at the protrusion tips may be important for elongation and to maintain the ultrastructure of stereocilia (Rzadzinska et al., 2005; Salles et al., 2009; Merritt et al., 2012; Mecklenburg et al., 2015; Ebrahim et al., 2016; Liu et al., 2016). Loss of both MYO3A and MYO3B in mice resulted in a severe deafness phenotype, as well as disrupted stereocilia structure and morphology (Lelli et al., 2016). In these studies, stereocilia were abnormally long and displayed an increase spacing between rows. When MYO3B alone was absent, there was little to no overall change in stereocilia structure (Ebrahim et al., 2016). Conversely, loss of MYO3A caused age-dependent hearing loss similar to the DFNB30 phenotype found in humans (Ebrahim et al., 2016; Lelli et al., 2016). Interestingly, in mice that were MYO3B^–/–^, MYO3A-PNcKO (i.e., contained MYO3A expression during post-natal development, but eliminated upon maturation), there was no deficit seen in stereocilia structure or function, suggesting class III myosins are crucial during development (Lelli et al., 2016). This same study found that both ESPN and MORN4 were able localize at stereocilia tips in the absence of MYO3A/B, suggesting that class III myosins are not required to transport these proteins to the tips. Overall, MYO3B appears to be able to partially compensate for the loss of MYO3A in DFNB30, while class III myosins appear to be most important during development.

Many MYO3A hearing loss mutations have been identified via genetic screens, while two dominant mutations, G488E and L697W, have been studied in depth (Grati et al., 2016; Dantas et al., 2018). G488E was shown to reduce ATPase activity but increase the *in vitro* motility of MYO3A (Grati et al., 2016). Interestingly, G488E failed to tip localize in the filopodia of COS-7 cells, though it did tip localize to stereocilia tips in organotypic inner ear hair cell cultures. We speculated that the G488E mutant may be unable to walk to the filopodia tips because of its disrupted ATPase cycle and or duty ratio, but in the stereocilia interactions with other binding partners may allow the mutant to translocate to the tips (e.g., as a complex with endogenous WT MYO3A). The L697W mutant was found to decrease both ATPase activity and *in vitro* motility, however, it did not have a defect in tip localization in COS-7 cells (Dantas et al., 2018). Interestingly, L697W was able to outcompete WT MYO3A for localization to protrusion tips in the presence of ESPN-1, while filopodia were overall shorter and fewer in number in the presence of the mutant. We proposed that L697W may be effective at localizing to the protrusion tips, and even more efficiently in the presence of ESPN-1, but its disrupted motor may prevent it from performing its tip associated functions important for length regulation. Further studies of the detailed ATPase mechanism of the G488E and L697 mutants will be extremely important as we attempt to relate the specific motor properties (e.g., duty ratio and attached lifetime) of MYO3A to its function in actin protrusions.

**FIGURE 4:**
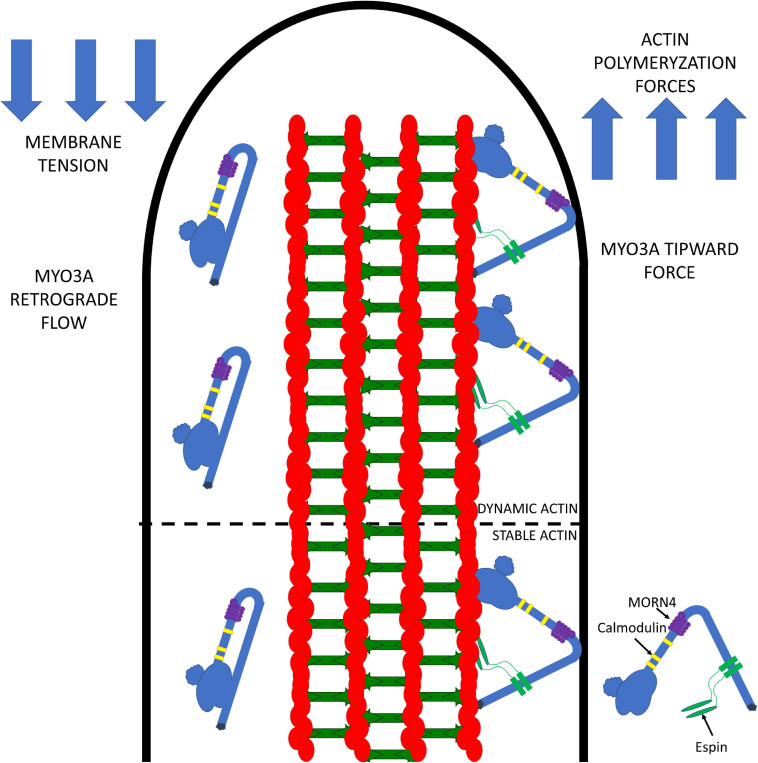
Working model for MYO3A function in inner ear hair cell stereocilia. MYO3A walks toward the plus end of actin protrusions utilizing its motor and actin binding tail domains and can dock at the tips by an unknown mechanism. The tip localized MYO3A can produce an ensemble force that works together with the actin polymerization forces to combat membrane tension, which can result in protrusion elongation or retraction (depending on the balance of forces). The amount of tip-directed force produced by MYO3A motors at the protrusion tips is impacted by the number of motors present, as well as their duty ratio and intrinsic force producing abilities. Examining how MYO3A motor-based forces are altered by physiological factors (e.g., phosphorylation and protein-protein interactions) or disease associated mutations (e.g., deafness mutations) will be the focus of future investigations that will further test this model.

Our current model for the role of MYO3A in actin protrusions is summarized in Figure 4. We propose that MYO3A walks toward the plus-ended actin protrusion tips and docks at the tips by some unknown mechanism (e.g., membrane binding and/or protein-protein interactions). The steady-state concentration of MYO3A at the tips is at least partially dependent on autoregulation by the kinase domain. We propose that the tip localized MYO3A can produce a plus-end directed force that along with actin polymerization forces can elongate the actin protrusion. The plus-end directed forces can combat the membrane tension to result in a net elongation of the protrusion in a mechanism similar to that proposed for myosin I and myosin X, whereby the motor is able to link the plasma membrane and actin cytoskeleton while exerting force (Nambiar et al., 2010; Watanabe et al., 2010; He et al., 2017). We predict that the turnover of MYO3A at the tips is at least partially controlled by the myosin duty ratio and steady-state actin affinity, since the higher actin affinity of MYO3A compared to MYO3B correlates with its more efficient tip localization (Raval et al., 2016). We also predict that the rate of elongation may be controlled by MYO3A motor activity, and likely correlates with *in vitro* actin gliding velocity since multiple MYO3A motors likely work together at the protrusion tips to generate force. The balance of forces at the tips likely controls the average length of the actin protrusion. For example, if membrane tension becomes greater than the motor and actin polymerization forces, the protrusion will undergo a net retraction. Overall, we hypothesize that the motor properties of MYO3A are critical for its role in protrusion length maintenance, and thus deafness mutations that alter the duty ratio, force production, or actin gliding properties will specifically disrupt the length regulation function of MYO3A.

Does MYO3A work together with MYO15 to regulate stereocilia length? We predict that MYO15 may perform a similar function by generating a protrusive force at the stereocilia tips to combat membrane tension and since it is a faster motor it may dominate the protrusion elongation rate. MYO3A is a slower motor that may function as a brake to prevent over-elongation of the stereocilia. This hypothesis is consistent with the mouse model results that demonstrate shorter stereocilia in the absence of MYO15 (Probst et al., 1998) but over-elongated stereocilia in the absence of MYO3A (Lelli et al., 2016). Although we have focused on the more well characterized MYO3A our model is also likely consistent with MYO3B playing a similar role at the protrusion tips.

## Summary and Discussion

There are several open questions related to the function of myosin motors in the stereocilia and other similar actin-based protrusions. One question is related to cargo transport within the stereocilia and the requirement for myosin motors to be processive to function as cargo transporters. Stereocilia-associated myosins were found to have a wide range of duty ratios and motility speeds and direct measurements of processive motion in single molecule motility assays with myosins thought to function in transport (e.g., MYO3A and MYO15A) are lacking. Nearly all of the stereocilia-associated myosins were found to have membrane binding motifs, which could be crucial for localization within a specific region of the stereocilia. Membrane binding could also be important for docking at the stereocilia tips and forming a link between the membrane and actin bundle that is necessary for elongation. Finally, it is unclear how different classes of myosin motors work together to function in the stereocilia. For example, MYO3A and MYO15A may work together to elongate the stereocilia and if so, how does this impact the overall rate of elongation since MYO3A slides actin filaments fivefold slower than MYO15A in the *in vitro* motility assay. In addition, MY07A and MYO1C may work together to mediate tension of the MET channel during adaptation. Future studies that will examine the motor properties of stereocilia-associated myosins both with *in vitro* biophysical approaches, cell biological studies, and *in vivo* transgenic mouse models will provide a clearer picture of the role of each myosin motor in the stereocilia.

## Author Contributions

JC and CY wrote the manuscript. LG provided the editorial input. All authors contributed to the article and approved the submitted version.

## Conflict of Interest

The authors declare that the research was conducted in the absence of any commercial or financial relationships that could be construed as a potential conflict of interest.
